# Paediatric Medicinal Formulation Development: Utilising Human Taste Panels and Incorporating Their Data into Machine Learning Training

**DOI:** 10.3390/pharmaceutics15082112

**Published:** 2023-08-09

**Authors:** Okhee Yoo, Britta S. von Ungern-Sternberg, Lee Yong Lim

**Affiliations:** 1 Pharmacy, School of Allied Health, University of Western Australia, Perth, WA 6009, Australia; okhee.yoo@uwa.edu.au; 2 Perioperative Medicine Team, Perioperative Care Program, Telethon Kids Institute, Perth, WA 6009, Australia; britta.regli-vonungern@uwa.edu.au; 3 Department of Anaesthesia and Pain Medicine, Perth Children’s Hospital, Perth, WA 6009, Australia; 4 Division of Emergency Medicine, Anaesthesia and Pain Medicine, The University of Western Australia, Perth, WA 6009, Australia

**Keywords:** taste panels, artificial neural networks (ANNs), paediatric drug formulations, taste masking, taste assessment, paediatric formulation development, machine learning

## Abstract

This review paper explores the role of human taste panels and artificial neural networks (ANNs) in taste-masking paediatric drug formulations. Given the ethical, practical, and regulatory challenges of employing children, young adults (18–40) can serve as suitable substitutes due to the similarity in their taste sensitivity. Taste panellists need not be experts in sensory evaluation so long as a reference product is used during evaluation; however, they should be screened for bitterness taste detection thresholds. For a more robust evaluation during the developmental phase, considerations of a scoring system and the calculation of an acceptance value may be beneficial in determining the likelihood of recommending a formulation for further development. On the technological front, artificial neural networks (ANNs) can be exploited in taste-masking optimisation of medicinal formulations as they can model complex relationships between variables and enable predictions not possible previously to optimise product profiles. Machine learning classifiers may therefore tackle the challenge of predicting the bitterness intensity of paediatric formulations. While advancements have been made, further work is needed to identify effective taste-masking techniques for specific drug molecules. Continuous refinement of machine learning algorithms, using human panellist acceptability scores, can aid in enhancing paediatric formulation development and overcoming taste-masking challenges.

## 1. Introduction

The development of acceptable paediatric medicinal products is required to counter the widespread and potentially harmful practice of off-label use of medicinal products that are licensed only for adults yet prescribed for paediatric patients. In a recent large consumer priority setting in Australia, more child-friendly medications and formulations were amongst the top 10 research priorities for paediatric anaesthesia and perioperative medicine [1]. Despite regulatory incentives in the United States and Europe to promote paediatric formulation development, progress is hampered by challenges including the need to address dose flexibility, swallowability, palatability, and the diverse physiological developmental stages encountered in the paediatric population. Peroral minitablets [2], microparticles [3], granules [4], liquid formulations, and scored chewable tablets [5,6] have been proposed to address dose flexibility and provide ease of swallowability. However, given that one in four active pharmaceutical ingredients (APIs) have an intensely bitter taste and these formulations often result in drug interactions with taste receptors, palatability remains a significant challenge as young children are highly sensitive to bitter taste [7]. This issue is especially prevalent for drugs required to be administered at high doses and frequent intervals, for example, anti-infectives, where the problem is further compounded by the high number (40%) of anti-infective APIs having an objectionable taste [7]. 

Achieving effective taste masking of paediatric medicinal formulations is therefore desirable, yet not always attainable through standard experimentation methods. From a quality by design (QbD) perspective, effective taste masking of unpalatable APIs is a critical quality attribute (CQA) that must be considered during formulation development in order to achieve a quality target product profile. As such, it is imperative that the CQA of palatability is built into the developmental process. Unfortunately, there is no single ideal method for taste evaluation of medicinal formulations during the development phase. Ideally, a taste evaluation of paediatric formulations should be conducted in the target paediatric population; however, ethical, and practical, considerations make this unfeasible, particularly for novel APIs. Consequently, various alternatives, such as in vitro drug dissolution/release, e-tongue, and the rodent aversion model, have been utilised as surrogates. There are, however, limitations in translating the data derived from these methods into formulation development [8,9]. 

The aim of this review article is to summarise the limitations of the surrogate methods of taste evaluation, advocate a practical implementation of appropriate human taste panels to evaluate the palatability of paediatric formulations during the preclinical pharmaceutical development phase, re-examine the concept of acceptability, and recommend the use of machine learning for the future of palatable paediatric formulation development.

## 2. In Vitro Models

Surrogate in vitro drug dissolution assessment involves measuring the percentage of drug load released at specified time points from a formulation into simulated saliva. The use of traditional in vitro dissolution data to evaluate the taste acceptance of a medicinal product is simple, cost-effective, and convenient. However, this method is not able to provide the threshold concentration for bitterness detection of a drug in the target population. It also does not consider the size of the drug load in the formulation, and thus would not be able to differentiate taste differences generated by a 10% release of a 5 mg drug dose and a 10% release of a 500 mg drug dose. Additionally, the in vitro dissolution data do not account for variations in bitterness intensity between drugs or the role of excipients in modulating the organoleptic properties of the formulation. Furthermore, there is no published consensus on the methodology regarding sampling time points, type, volume, and agitation of the simulated saliva dissolution medium for in vitro drug dissolution studies to generate reliable surrogate taste scores [10,11]. 

E-tongue technology employs a range of flavour sensors to detect the five basic tastes: bitterness, sweetness, saltiness, sourness, and umami. These sensors operate by measuring changes in the membrane’s chemical potential when immersed in a liquid medium, and the resulting signals are converted into a chemical pattern [12]. Lack of capacity to identify non-ionisable drugs in the sample medium is a constraint of the e-tongue technique [13]. This stands in contrast to the human tongue’s ability to detect the taste of non-ionisable molecules [14]. Additionally, the e-tongue is limited to the evaluation of solutions. When the formulation under study is a solution, the sensors can be directly immersed in the formulation to measure its taste. For formulations that are not solutions, which account for the majority of new paediatric formulations, in vitro drug dissolution experiments have to be conducted and the e-tongue is employed to analyse the drug released from the formulation into the dissolution medium. Thus, the e-tongue is not capable of examining the role of excipients, nor measuring the texture and acceptability of the original formulation, resulting in its data not always able to be correlated with human taste data [15]. However, e-tongue technology has advanced in recent years, and biological electronic tongues (BioETs) now include bioactive materials such as receptors, cells, tissues, and other systems that aim to replicate the biological processes to more closely mimic human taste perception [16]. In one study, a BioET has been shown to provide a high degree of sensitivity and specificity to the taste of a variety of medium- and long-chain fatty acids, including lauric acid, linoleic acid, and docosahexaenoic acid [17]. Also, since BioETs are not restricted to only testing solution formulations, the influence of excipients on taste modification could potentially be detected using BioETs, in conjunction with artificial neural network (ANN) algorithms. Thus, while BioETs are a fascinating development, an in-depth knowledge of receptor cells that specialise in detecting distinct tastes or textures is crucial for determining overall taste acceptability using this technique. It is also yet to be validated against human gustatory data.

## 3. In Vivo Animal Models

The rodent-based brief-access taste aversion (BATA) assay has been proposed as an alternative to human taste panels and e-tongues for evaluating the palatability of APIs. The BATA method uses a lickometer to measure the frequency of licks made by mildly water-deprived rodents when presented with API tastant solutions at a range of concentrations, with low licking rates relative to the blank vehicle and/or water suggestive of aversive tastes [18]. While this method enables the generation of a comprehensive concentration–response curve for lick rates within a short timeframe and with minimal animal usage, a major concern is the ethical treatment of animals as they are unable to expel the aversive tastants. The experiments are performed under highly controlled environments as the animals’ licking behaviour is readily affected by noise and light distractions. Rodents also typically exhibit heightened sensitivity to bitterness tastes compared to humans, which may not allow for direct data translation, and the necessity for ethical approvals may prolong product development timelines. 

Fish or flies have also been used to evaluate the deterring effects of tastants [19,20], but there is no published method for the taste evaluation of medicinal formulations using these animal models. Taste scores generated using animal models have to be translated to provide equivalent human data, and while there are tools to translate taste scores from various non-human models to human equivalents [15,21], it is difficult to correlate an animal aversive response to the nuances of human taste data. 

## 4. Human Taste Panel

Human taste panels are regarded as the gold standard for the taste evaluation of products designed for humans [21]. Ideally, human taste panels should be applied as the reference point for evaluating taste at multiple phases of paediatric formulation development, both before and during product creation, and also during the final assessment of the product through clinical trials [22,23,24]. Despite their desirability, establishing an appropriate human taste panel for all stages of development for a paediatric medicinal formulation has not been extensively discussed within the pharmaceutical science community. 

Consensus-driven human taste panel data will also be important heading into the future. Currently, the process of developing an effective taste-masked formulation for an unpalatable API is not efficient, predominantly relying on a trial-and-error method. An approach that utilises ANN algorithms to forecast an optimal taste-masking formulation for a specific drug molecule in a target dosage form could result in considerable cost efficiency. The establishment of an appropriate ANN requires human taste panels involved in evaluating paediatric formulations to use a unified scoring system agreed upon by the pharmaceutical industry. In the next sections, we delve into the qualifications required for the taste panellists, suggest a novel scoring system, and discuss their function in serving as the training dataset for an artificial intelligence-driven taste-masking formulation selection.

### 4.1. Ethical Consideration

The ideal taste panellist for paediatric formulations would be a child. However, there are a range of practical and ethical issues that make it highly challenging to enrol children for taste trials of medicines [25]. There are much higher levels of responsibility in areas like confidentiality, legal consent, and absolute risk assessment, and stricter ethical codes of conduct have to be put in place when children are involved [26]. It is also much more difficult to recruit adequate numbers of paediatric participants, and involvement in trials with unpleasant stimuli can be very distressing for young patients and their parents. Furthermore, when young children (<5 years old) are the target group, instructions for the taste evaluation procedure can be challenging to convey and compliance with these instructions may be suboptimal. The evaluation of novel APIs presents further difficulties, as safety data may not yet be fully available, leading clinicians to be hesitant to be involved while parents are reluctant to provide consent. Nonetheless, if the continuous evaluation of taste during the medicinal product development phase is crucial, then feedback from the relevant population is indispensable in determining acceptable taste profiles. In the QbD approach, this can only be accomplished through the incorporation of validated taste evaluation protocols in the design of experiments (DoE).

### 4.2. Identifying Suitable Panellists

Young adult panellists capable of providing independent consent may be a practical alternative to circumvent the ethical barriers associated with using child panellists for the taste evaluation of medicinal products. However, the correlation between adult and child gustatory experiences remains debatable, with concern centring around taste perception, particularly aversion to bitterness taste, which is significantly different between young children and adults [27]. Nonetheless, the perception that sensitivity to bitterness in children only becomes comparable to that of adults during mid-adolescence has not been verified.

In the field of sensory evaluation, normative values for gustatory sensitivity have been established for both adults and, more recently, children. The data were obtained using “Taste Strips”, which are a validated gustatory test for four taste endpoints: sweet, sour, salty, and bitter. Initially, the taste strips were administered following a predefined pseudo-randomised procedure for adults aged 18–87 years [28]. A total of four different tastants were tested, with each tastant administered at four taste-differentiable concentrations. The gustatory function was provided by a cumulative score, derived from tallying the tastes correctly identified. The normative value, defined as the 10th percentile of the study values, was determined. A similar study was also recently conducted to determine the normative value for children aged 6–15 years [29]. Although the participants in this study included those up to 17 years of age, the small sample size for the 16- and 17-year-olds precludes these participants in the final data analysis. Comparisons of the datasets in the two studies indicate that the human taste function reaches maturity around the age of 10 years, with maturity occurring slightly faster in girls. In children aged 6–15 years, taste scores for sweet, salty, and bitter flavours, as well as the total taste score, increased with age. Conversely, in adults aged 18–87 years, the taste function decreased with advancing age above 40 years old. It is hypothesised that the regeneration of taste buds every 1 to 2 weeks leads to a general ability to maintain taste sensitivity with increasing age in most people; however, in individuals older than 40 years, the taste bud regeneration becomes unstable, leading to less reliable taste perception. This suggests that adults under the age of 40 years have similar taste discrimination scores to children aged between 10 and 15 years, and they may have a more refined ability to distinguish tastes compared to children younger than 10 years old. On this basis, the recruitment of adults aged 18–40 years as taste panellists could provide a practical solution to providing ongoing taste assessment of paediatric formulations during the development phase.

### 4.3. Taste Panellists for Medicinal Formulations: Is Training Necessary?

Sensory evaluation has traditionally been categorised into two distinct areas: analytical tests, which are commonly utilised in the food industry to objectively assess a range of sensory attributes of products, and hedonic tests, which evaluate consumer acceptance or preference for a product [30]. The nuanced taste characteristics of a paediatric formulation is not as crucial as those of a food product; in fact, highly attractive and palatable medicines may cause children to overdose. What is more critical for a paediatric medicinal formulation is to assess the threshold at which its taste becomes acceptable. Hence, hedonic tests that evaluate the acceptance or tolerability of the formulation are adequate as the primary sensory evaluation during the paediatric formulation development phase. 

In line with this, conventional sensory panels comprising experts who are highly trained in providing detailed taste descriptions, which are often required in ensuring quality assurance for food, perfumes, wines, and beer [31,32,33], may not be necessary for paediatric formulation development. However, numerous studies have indicated that trained assessors do outperform consumers, primarily due to their familiarity with experimental procedures used for sample evaluation [34] and their ability to articulate their taste perceptions [35]. Recent research has shown that even brief familiarisation can be helpful to enhance the consumer performance in analytical taste tests [36]. Thus, while the training to provide detailed taste descriptions is not essential for the taste evaluation of paediatric medicinal formulation, a discriminative deterrence for a taste, e.g., bitterness, that is relevant to the acceptability of the formulation will be required of the taste panellist. This ability to distinguish between samples can be improved with familiarisation, and untrained panels can yield results comparable to those produced by trained panels when provided with appropriate reference tastant samples [30].

Most medicinal formulations with objectionable sensory qualities contain a poorly taste-masked bitter-tasting drug. Bitterness is an aversion taste; however, there is genetic variation in how bitterness is perceived as it is detected by a group of receptors with a high degree of polymorphism [37]. Fox, in 1932, had already recognised through blind taste trials of phenylthiocarbamide that some people are extremely sensitive to bitter tastes while others, the nontasters, perceived the same agent to have no or an only slightly bitter taste [38]. Nontasters have been observed to become more sensitive to the bitter taste of quinine hydrochloride when the agent is applied to the back of tongue where the bitterness receptors are located [39]. To minimise variation in bitterness taste perception in the taste panel, a preliminary study using solutions containing quinine at various concentrations above its threshold bitterness perception concentration to screen extreme outliers in bitterness taster panels is encouraged. However, with nontasters making up 30% of the general population [40], it may be argued that at least one nontaster should be included in the taste panel to provide more representative scores for the medicinal formulation. 

It is also helpful to identify and address the challenges encountered by taste panellists. During the DoE developmental stage where multiple formulation samples can be prepared, panellists may have to taste a multitude of samples in a single day. This can lead to sensory fatigue, causing uncertainty when panellists are comparing samples that were presented early in the session with those presented at the end of the session. Emotional factors can also impact the results, although this limitation can be overcome by using a standard reference sample, similar to the method employed by Teillet et al. [41], to provide similar baselines for different taste sessions. A known concentration of quinine solution can serve as a reference product. Another obstacle in discriminatory taste evaluation is the persistence of an aftertaste, which is not uncommon among bitter APIs. Water and plain crackers are often offered to taste panellists to neutralise residual tastes; however, this approach is not effective for APIs that leave a lingering taste for several hours. Given the time constraints of evaluation sessions, a washout period of several hours between samples is impractical. Therefore, there is a pressing need for suitable interventions for taste evaluation of formulations with APIs that leave a prolonged lingering residual taste.

### 4.4. Towards a Unified Taste Scoring System in Paediatric Medicinal Formulations

Scores or scales are commonly used to rate the taste of paediatric medicinal formulations, with most studies employing numeric systems and some utilising categorical scoring methods. However, the interpretation of these scales can be inconsistent, making inter-study comparisons difficult. One taste-scoring system for evaluating oral medicinal formulations is the visual analogue scale (VAS), where a 10 cm scale has 10 score points, with 0 indicating the best, and 10 the worst, taste. The scale is further divided into four categories: “excellent” (scores of 0–2), “good” (3–5), “acceptable” (6–8), and “poor” (>8) [24]. Orubu et al. used a computerised questionnaire, where participants rated aversion and grittiness on continuous and categorical scales, respectively. Aversion was measured on a 100 mm scale ranging from “not aversive” (0) to “very aversive” (100). Grittiness was assessed using a 5-point Likert scale ranging from “not gritty” (1) to “gritty” (5). Additionally, participants indicated whether they found the sample acceptable as a medicine with a simple “yes” or “no.” [22] Our team used the five-point facial hedonic scale for both children and young adult taste panels, with options ranging from “dislike very much” on the left to “like very much” to the right, and children were further asked to provide a simple “yes” or “no” to indicate whether they would be willing to take the medicine again [5,6]. These scoring systems vary in complexity, measurement scales, and target populations. The VAS and computerised questionnaire use continuous scales for rating taste, while the facial hedonic scale employs categorical rankings. Table 1 presents examples of the methodologies, along with their references, that are used for evaluating the acceptability of oral paediatric formulations.

The acceptability of a medicinal product is a complex concept that is not likely to be quantifiable by a single taste score. In clinical taste trials involving children, in particular children under 5 years of age who may not fully understand the purpose of the trial, we have observed that factors such as pain, difficult relationships with caregivers, and the individual’s anxiety can adversely influence their taste score, leading, for example, to a child giving a poor taste score who at the same time would request to have another trial tablet. Some studies have attempted to address this by employing a questionnaire that covers multiple aspects contributing to palatability rather than a single parameter of measure, however; this still does not address the interpretation of acceptability. 

In a 2003 issue of the *Harvard Business Review*, Reichheld introduced the concept of “the one number you need to grow”: the net promoter score [43]. Companies can bypass complex and ambiguous satisfaction measures by simply asking customers if they would recommend the company to a friend or colleague [43]. According to this idea, one only needs to ask the target customers a single question: “How likely is it that you would recommend our company to a friend or colleague?” The greater the number of “promoters” a company has, the more significant would be its growth. To determine the net promoter score, the customers’ responses based on a 0 to 10 rating scale are categorised into “promoters” (9–10 rating—extremely likely to recommend), “passively satisfied” (7–8 rating), and “detractors” (0–6 rating—extremely unlikely to recommend), and the percentage of detractors is subtracted from the percentage of promoters. Companies with exceptional loyalty will achieve net promoter scores ranging from 75% to over 80% [44].

The application of the net promoter score is prevalent in the business world, but this concept has yet to be applied for the assessment of palatability and therapeutic compliance of medicinal products. Instead, published studies assessing paediatric formulations have largely referenced the taste assessment methodologies of the food industry. We know from clinical trials involving children that the acceptability of a paediatric medicine is influenced by factors other than taste. Instead of trying to integrate all these factors into an acceptability index, it may well be adequate to simply ask a single, comprehensive question that encapsulates the overall acceptance of the medicinal product. The goal is to develop medicinal products that are acceptable, effective, and safe without causing the child to refuse to take it. Thus, posing straightforward queries such as “How probable is it that you would recommend this medicine for your child or others?” or “How likely would you be to take this medicine if you were ill?”, combined with a data analysis method that consolidates rejection, indecisiveness, and acceptance into a single score, could provide a more accurate and persuasive representation of medication acceptance in the pharmaceutical field. In essence, the question here is whether it is possible to apply the concept of the “one number for ACCEPTANCE” to the evaluation of paediatric medicinal product evaluations.

As a worked example, consider Table 2 and Table 3, which display the hypothetical taste scores for four formulations obtained from 10 participants. The question posed for Table 2 is “On a scale of 0 to 10, where 0 represents strong dislike and 10 represents strong liking, what is your rating for the taste of the formulation?”, while that for Table 3 is “Would you be willing to take the formulation again if required?” From the data in Table 2, it is evident that formulation B has the highest average taste score and is likely the preferred option. However, it is not clear whether an average score of 5.9 is adequately differentiated from 7.3, and whether a score of 7.3 is sufficient to recommend that the manufacturer progress with formulation B with confidence that the formulation would not elicit refusal from children. For Table 3a, the participants’ scores for “willingness to take the formulation again” are stratified according to the Reichheld’s business model. Using this approach, both formulations C and D have negative scores, providing a clearer indication that they have a low likelihood of being promoted. Of the four formulations, only formulation B has a positive score, suggesting that it is likely to be promoted. However, using the business model, the score for formulation B is not adequate to progress as the loyalty concept requires a net score of 75–80%. It might be argued that the classification boundaries and net promoter score could be less stringent for medicinal formulations since the aim is not to develop medicines as delicious treats. Instead, a certain level of positivity would be sufficient. The downward adjustment of the classification boundaries for the participants’ responses in Table 3b results in net promoter scores that are less discriminatory than those in Table 3a. 

In summary, it is compelling to have the net promoter score provide a single value, which may be more appropriately named “medicine acceptance score”, to provide a more robust representation of acceptance of a paediatric medicinal product compared to current published scoring systems. For this approach to be adopted for paediatric medicinal formulation assessment, it is essential to establish classification boundaries and a net promoter score that the pharmaceutical community agrees upon. 

## 5. Optimising Taste Masking with Artificial Neural Networks

The implementation of the QbD approach to medicinal product development has led to the DoE being one of the most frequently utilised tools by pharmaceutical scientists [45]. However, the DoE, which relies on mathematical modelling, may not be as powerful a tool as artificial neural networks (ANNs), a machine learning approach inspired by the human brain. ANNs form the foundation of deep learning algorithms, and they have the potential to serve as valuable experimental tools for optimising and predicting desired product profiles. In contrast to DoE, ANNs can simultaneously model a large number of variables and establish intricate relationships between dependent and independent variables. Furthermore, ANNs can manage multiple outputs and model unstructured data, whereas the DoE mathematical models might be too simplistic to describe complex input-output relationships. In the field of pharmaceutical development, ANNs have been employed within the design space for predicting responses such as dissolution [46,47] and for optimising process parameters [48], but they have not yet been used to predict optimised formulation designs for an unpalatable API. 

Moreover, with DoE, the initial design of a taste-masked paediatric formulation still largely depends on a process of trial and error, supported by the published literature, researcher expertise, and previous experiences, with consumer feedback becoming an increasingly significant component of this research process. Selecting a taste-masking formulation is a complex task, and there is as of yet no universal taste-masking technology that works for all APIs. There are numerous methods for masking the bitterness of APIs, including the addition of agents to mask taste (flavours and sweeteners) or inhibit the bitterness taste receptor, physical barrier methods (coating, granulation, emulsification, gelation), and chemical methods (tasteless prodrugs and ion-exchange complexation). The chosen method depends on multiple factors, such as the bitterness threshold concentration of the API, API–excipient interactions, physicochemical properties of the API, API dose to be administered, and the API load released from the formulation into the oral cavity. The first step in QbD involves designing a formulation that considers all the above factors. The research required for this can be resource-heavy, time-consuming, and at the same time uncertain, as the chosen formulation design may not successfully mask the API bitterness to an acceptable level, making it a costly endeavour. 

The diversity of bitter molecules can make it challenging to predict whether a compound will taste bitter based on its chemical structure. However, researchers have developed machine learning classifiers to resolve this, although the bitter taste prediction remains limited to small molecules, bitter receptors for small molecules [49], and peptides [50]. For instance, BitterDB is a database of compounds reported to taste bitter to humans and some animals [49]. BitterPredict is a machine-trained model using data from BitterDB and non-bitter compounds from the literature, and claims to achieve high accuracy in classifying unknown compounds as tasting bitter or non-bitter [51]. Although useful for classifying large compound sets, this dichotomous classification is less helpful for medicinal formulation development where different taste-masking methods may be required depending on the level of API bitterness intensity. Another tool, BitterIntense, is a machine learning classifier that identifies molecules as “very bitter” or “not very bitter” based on chemical structure, boasting over 80% accuracy on several test sets [7]. More recently, a web-based database was created specifically for APIs prescribed for children [52]. This publicly available and electronically searchable database allows users to input the Simplified Molecular-Input Line-Entry System (SMILES) files of the main drug molecules to predict the taste of the resultant oral medicines [52]. VirtualTaste is yet another free-to-use web-based platform. Unlike the other databases, it is designed to predict three taste endpoints—sweet, bitter, and sour—of individual compounds based on molecular fingerprinting determined by the machine learning algorithms built into the platform using published human data. The computational model claims to have an accuracy exceeding 88% and to provide a balance between specificity and sensitivity [53]. 

These databases are designed to predict the taste, in particular the bitterness, of individual compounds. They do not provide protocols for the taste assessment of complex admixtures of excipients and APIs typically present in medicinal formulations. However, advancements in knowledge and machine learning technologies over the past decade may make it possible for researchers to use machine learning to identify patterns in the relationship between molecules and effective taste-masking technologies. Machine learning can integrate numerous variables and uncover hidden correlations that contribute to specific taste experiences and may become proficient at discerning bitterness and its intensity in complex medicinal formulations. A critical requirement lies in having a dataset for the most effective technique for masking the bitterness of specific molecules. The use of machine learning to select formulation designs depends on acquiring knowledge from existing experimental data and previous experiences, and then making accurate predictions for new applications using the learned information from training datasets. Indispensable data for this training dataset include the concept of acceptance, which may well be provided by the net promoter score obtained from human taste panels, as outlined in the previous section. Validated and consistent acceptability scores from human taste panels together with the formulation details (excipients, manufacture processes, and taste-masking platform) are considered vital for advancing paediatric formulation development to the next level.

## 6. Conclusions

In conclusion, the ideal taste panellists for paediatric formulations would be children; however, due to various ethical, practical, and regulatory challenges, young adults are often employed as substitutes. Taste sensitivity matures around the age of 10 and declines with age, making adults aged 18–40 potentially more suitable representatives for evaluating paediatric formulations. When it comes to training, it may not be necessary for taste panellists to become expert tasters as long as a reference product is used and taste panellists are screened for individual bitterness taste detection thresholds. A unified taste scoring system for paediatric formulations has yet to be established, but adopting a simplified approach, like asking a single question regarding the likelihood of recommending the medicine, could provide a more accurate representation of medication acceptance. This approach, combined with establishing a compliance value that the pharmaceutical community can agree upon, could lead to a more consistent and effective evaluation system for paediatric formulations. 

Artificial neural networks (ANNs) are an untapped resource in the field of taste-masking optimisation, offering a more comprehensive approach compared to the traditional design of experiments (DoE). With the ability to model intricate relationships between numerous variables, ANNs have the potential to better predict and optimize desired product profiles. The challenge of taste masking in paediatric formulations can be addressed by employing machine learning classifiers to predict bitterness and its intensity. While significant strides have been made in this area, further advancements are required to identify the most effective taste-masking techniques for specific molecules. By utilizing human panellist acceptability scores and continually refining machine learning algorithms, researchers can work towards improving paediatric formulation development and overcoming taste-masking challenges.

## Figures and Tables

**Table 1:** Examples of methodologies utilised for evaluating the acceptability of oral paediatric formulations.

Tools Used	Scales Used	Ref.
Facial hedonic scale	5 faces	[5,6]
Facial hedonic scale	7 faces	[42]
Visual analogue scales (VASs)	10 cm scale: “excellent” (0–2), “good” (3–5), “acceptable” (6–8), and “poor”	[24]
Visual analogue scales (VASs)	“Not aversive” (0) to “very aversive” (100)	[22]
Likert scale	6 levels from “Not bitter” (1) to “extremely bitter” (6)	[23]

**Table 2:** Hypothetical taste scores from 10 participants for the evaluation of four medicinal formulations. Mean and median scores are based on a 0–10 taste-rating scale (0—strongly dislikes, 10—strongly likes).

	A	B	C	D
	2	6	3	1
	2	6	4	2
	3	6	4	2
	3	7	5	5
	3	7	6	5
	6	7	6	5
	6	7	6	5
	7	8	7	8
	7	9	8	8
	9	10	10	9
Mean Median	4.8	7.3	5.9	5
	4.5	7	6	5

**Table 3:** Hypothetical acceptance scores from 10 participants for “Willingness to Re-take Formulation” for 4 medicinal formulations. (**a**) Classification of responses: distractors (0–6, peach), passives (7–8), and promoters (9–10, green). (**b**) Classification of responses: distractors (0–5, peach), passives (6–7), and promoters (8–10, green).

(a)	A	B	C	D
	2	6	3	2
	2	7	3	3
	2	7	5	3
	3	7	7	5
	5	8	8	5
	6	8	8	5
	6	8	8	6
	7	8	8	8
	8	9	9	9
	10	10	10	9
Distractor	70	10	30	70
Promoter	10	20	20	20
Net Promoter Score	−60	10	−10	−50
**(b)**	**A**	**B**	**C**	**D**
	2	6	3	2
	2	7	3	3
	2	7	5	3
	3	7	7	5
	5	8	8	5
	6	8	8	5
	6	8	8	6
	7	8	8	8
	8	9	9	9
	10	10	10	9
Distractor	50	0	30	60
Promoter	20	60	60	30
Net Promoter Score	−30	60	30	−30

## Data Availability

Data sharing not applicable.

## References

[1] Sommerfield A., Sommerfield D., Bell E., Humphreys S., Taverner F., Lee K., Frank B., von Ungern-Sternberg B.S. (2023). Consumer Research Priorities for Pediatric Anesthesia and Perioperative Medicine. Paediatr. Anaesth..

[2] Thomson S.A., Tuleu C., Wong I.C.K., Keady S., Pitt K.G., Sutcliffe A.G. (2009). Minitablets: New Modality to Deliver Medicines to Preschool-Aged Children. Pediatrics.

[3] Muoka L.C., Ross S.A., Mithu M.S.H., Nandi U., Douroumis D. (2021). Comparative Taste-Masking Evaluation of Microencapsulated Bitter Drugs Using Smartseal 30D and ReadyMix for Paediatric Dosage Forms. AAPS PharmSciTech.

[4] Neumann U., Whitaker M.J., Wiegand S., Krude H., Porter J., Davies M., Digweed D., Voet B., Ross R.J., Blankenstein O. (2018). Absorption and Tolerability of Taste-Masked Hydrocortisone Granules in Neonates, Infants and Children under 6 Years of Age with Adrenal Insufficiency. Clin. Endocrinol..

[5] Salman S., Tang E.K.Y., Cheung L.C., Nguyen M.N., Sommerfield D., Slevin L., Lim L.Y., von Ungern Sternberg B.S. (2018). A Novel, Palatable Paediatric Oral Formulation of Midazolam: Pharmacokinetics, Tolerability, Efficacy and Safety. Anaesthesia.

[6] Yoo O., Tang E.K.Y., Salman S., Nguyen M.N., Sommerfield D., Sommerfield A., Khan N., von Ungern Sternberg B.S., Lim L.Y. (2022). A Randomised Controlled Trial of a Novel Tramadol Chewable Tablet: Pharmacokinetics and Tolerability in Children. Anaesthesia.

[7] Margulis E., Dagan-Wiener A., Ives R.S., Jaffari S., Siems K., Niv M.Y. (2021). Intense Bitterness of Molecules: Machine Learning for Expediting Drug Discovery. Comput. Struct. Biotechnol. J..

[8] Scholes P. (2018). Removing the Bitter Taste from Drug Development. Pharm. Technol..

[9] Pein M., Preis M., Eckert C., Kiene F.E. (2014). Taste-Masking Assessment of Solid Oral Dosage Forms–A Critical Review. Int. J. Pharm..

[10] Keeley A., Teo M., Ali Z., Frost J., Ghimire M., Rajabi-Siahboomi A., Orlu M., Tuleu C. (2019). In Vitro Dissolution Model Can Predict the in Vivo Taste Masking Performance of Coated Multiparticulates. Mol. Pharm..

[11] Gittings S., Turnbull N., Roberts C.J., Gershkovich P. (2014). Dissolution Methodology for Taste Masked Oral Dosage Forms. J. Control. Release Off. J. Control. Release Soc..

[12] Guedes M.D.V., Marques M.S., Guedes P.C., Contri R.V., Kulkamp Guerreiro I.C. (2021). The Use of Electronic Tongue and Sensory Panel on Taste Evaluation of Pediatric Medicines: A Systematic Review. Pharm. Dev. Technol..

[13] Woertz K., Tissen C., Kleinebudde P., Breitkreutz J. (2011). Taste Sensing Systems (Electronic Tongues) for Pharmaceutical Applications. Int. J. Pharm..

[14] Choi D.H., Kim N.A., Nam T.S., Lee S., Jeong S.H. (2014). Evaluation of Taste-Masking Effects of Pharmaceutical Sweeteners with an Electronic Tongue System. Drug Dev. Ind. Pharm..

[15] Keating A.V., Soto J., Forbes C., Zhao M., Craig D.Q.M., Tuleu C. (2020). Multi-Methodological Quantitative Taste Assessment of Anti-Tuberculosis Drugs to Support the Development of Palatable Paediatric Dosage Forms. Pharmaceutics.

[16] Tian Y., Wang P., Du L., Wu C. (2022). Advances in Gustatory Biomimetic Biosensing Technologies: In Vitro and in Vivo Bioelectronic Tongue. TrAC Trends Anal. Chem..

[17] Lu Y., Huang Y., Li S., Zhang Q., Wu J., Xiong Z., Xiong L., Wan Q., Liu Q. (2017). Fat Taste Detection with Odorant-Binding Proteins (OBPs) on Screen-Printed Electrodes Modified by Reduced Graphene Oxide. Sens. Actuators B Chem..

[18] Soto J., Keeley A., Keating A.V., Mohamed-Ahmed A.H.A., Sheng Y., Winzenburg G., Turner R., Desset-Brèthes S., Orlu M., Tuleu C. (2018). Rats Can Predict Aversiveness of Active Pharmaceutical Ingredients. Eur. J. Pharm. Biopharm..

[19] Boyer B., Ernest S., Rosa F. (2013). Egr-1 induction provides a genetic response to food aversion in zebrafish. Front. Behav. Neurosci..

[20] Lvovskaya S., Smith D.P. (2013). A Spoonful of Bitter Helps the Sugar-Response Go Down. Neuron.

[21] Clapham D., Bennett J., Cram A., Discihnger A., Inghelbrecht S., Pensé-Lhéritier A.-M., Ruiz F., Salunke S., Schiele J., Soto J. (2022). Proposed Tool to Compare and Assess the Applicability of Taste Assessment Techniques for Pharmaceuticals. J. Pharm. Sci..

[22] Orubu S., Kendall R.A., Sheng Y., Tuleu C. (2022). Evaluating the Taste Masking Ability of Two Novel Dispersible Tablet Platforms Containing Zinc Sulfate and Paracetamol Reconstituted in a Breast Milk Substitute. Pharmaceutics.

[23] Li J., Li C., Zhang H., Gao X., Wang T., Wang Z., Zheng A. (2022). Preparation of Azithromycin Amorphous Solid Dispersion by Hot-Melt Extrusion: An Advantageous Technology with Taste Masking and Solubilization Effects. Polymers.

[24] Wang Z., Li J., Hong X., Han X., Liu B., Li X., Zhang H., Gao J., Liu N., Gao X. (2021). Taste Masking Study Based on an Electronic Tongue: The Formulation Design of 3D Printed Levetiracetam Instant-Dissolving Tablets. Pharm. Res..

[25] Conroy S., McIntyre J., Choonara I., Stephenson T. (2000). Drug Trials in Children: Problems and the Way Forward. Br. J. Clin. Pharmacol..

[26] McIntosh N., Bates P., Brykczynska G., Dunstan G., Goldman A., Harvey D., Larcher V., McCrae D., McKinnon A., Patton M. (2000). Guidelines for the ethical conduct of medical research involving children. Arch. Dis. Child..

[27] Mennella J.A., Bobowski N.K. (2015). The Sweetness and Bitterness of Childhood: Insights from Basic Research on Taste Preferences. Physiol. Behav..

[28] Landis B.N., Welge-Luessen A., Brämerson A., Bende M., Mueller C.A., Nordin S., Hummel T. (2009). “Taste Strips”—A Rapid, Lateralized, Gustatory Bedside Identification Test Based on Impregnated Filter Papers. J. Neurol..

[29] Van den Brink M., IJpma I., Fiocco M., Tissing W.J.E., Havermans R.C. (2022). Taste Function in Children: Normative Values and Associated Factors. Pediatr. Res..

[30] Ares G., Varela P. (2017). Trained vs. consumer panels for analytical testing: Fueling a long lasting debate in the field. Food Qual. Prefer..

[31] Moskowitz H.R. (1996). Experts Versus Consumers: A Comparison. J. Sens. Stud..

[32] Habschied K., Krstanović V., Mastanjević K. (2022). Beer Quality Evaluation—A Sensory Aspect. Beverages.

[33] Deubler G., Zhang C., Talavera M.J., Swaney-Stueve M. (2022). Sensory Evaluation in the Personal Care Space: A Review. J. Sens. Stud..

[34] Ishii R., Kawaguchi H., O’Mahony M., Rousseau B. (2007). Relating Consumer and Trained Panels’ Discriminative Sensitivities Using Vanilla Flavored Ice Cream as a Medium. Food Qual. Prefer..

[35] Chollet S., Valentin D. (2001). Impact of Training on Beer Flavor Perception and Description: Are Trained and Untrained Subjects Really Different?. J. Sens. Stud..

[36] Jaeger S.R., Beresford M.K., Hunter D.C., Alcaire F., Castura J.C., Ares G. (2017). Does a Familiarization Step Influence Results from a TCATA Task?. Food Qual. Prefer..

[37] Drayna D. (2005). Human Taste Genetics. Annu. Rev. Genom. Hum. Genet..

[38] Fox A.L. (1932). The Relationship between Chemical Constitution and Taste. Proc. Natl. Acad. Sci. USA.

[39] Sato T., Okada Y., Miyamoto T., Fujiyama R. (1997). Distribution of Non-Tasters for Phenylthiocarbamide and High Sensitivity to Quinine Hydrochloride of the Non-Tasters in Japanese. Chem. Senses.

[40] Tepper B.J., White E.A., Koelliker Y., Lanzara C., d’Adamo P., Gasparini P. (2009). Genetic Variation in Taste Sensitivity to 6-n-Propylthiouracil and Its Relationship to Taste Perception and Food Selection. Ann. N. Y. Acad. Sci..

[41] Teillet E., Schlich P., Urbano C., Cordelle S., Guichard E. (2010). Sensory Methodologies and the Taste of Water. Food Qual. Prefer..

[42] Thompson A., Reader S., Field E., Shephard A. (2013). Open-Label Taste-Testing Study to Evaluate the Acceptability of Both Strawberry-Flavored and Orange-Flavored Amylmetacresol/2,4-Dichlorobenzyl Alcohol Throat Lozenges in Healthy Children. Drugs RD.

[43] Reichheld F.F. (2003). The One Number You Need to Grow. Harv. Bus. Rev..

[44] Fisher N.I., Kordupleski R.E. (2019). Good and Bad Market Research: A Critical Review of Net Promoter Score. Appl. Stoch. Models Bus. Ind..

[45] Cook J., Cruañes M.T., Gupta M., Riley S., Crison J. (2014). Quality-by-Design: Are We There Yet?. AAPS PharmSciTech.

[46] Obeid S., Madžarević M., Krkobabić M., Ibrić S. (2021). Predicting Drug Release from Diazepam FDM Printed Tablets Using Deep Learning Approach: Influence of Process Parameters and Tablet Surface/Volume Ratio. Int. J. Pharm..

[47] Simões M.F., Silva G., Pinto A.C., Fonseca M., Silva N.E., Pinto R.M.A., Simões S. (2020). Artificial Neural Networks Applied to Quality-by-Design: From Formulation Development to Clinical Outcome. Eur. J. Pharm. Biopharm..

[48] Rodríguez-Dorado R., Landín M., Altai A., Russo P., Aquino R.P., Del Gaudio P. (2018). A Novel Method for the Production of Core-Shell Microparticles by Inverse Gelation Optimized with Artificial Intelligent Tools. Int. J. Pharm..

[49] Dagan-Wiener A., Di Pizio A., Nissim I., Bahia M.S., Dubovski N., Margulis E., Niv M.Y. (2019). BitterDB: Taste Ligands and Receptors Database in 2019. Nucleic Acids Res..

[50] Charoenkwan P., Yana J., Schaduangrat N., Nantasenamat C., Hasan M.M., Shoombuatong W. (2020). IBitter-SCM: Identification and Characterization of Bitter Peptides Using a Scoring Card Method with Propensity Scores of Dipeptides. Genomics.

[51] Dagan-Wiener A., Nissim I., Ben Abu N., Borgonovo G., Bassoli A., Niv M.Y. (2017). Bitter or Not? BitterPredict, a Tool for Predicting Taste from Chemical Structure. Sci. Rep..

[52] Bai G., Wu T., Zhao L., Wang X., Li S., Ni X. (2021). CBDPS 1.0: A Python GUI Application for Machine Learning Models to Predict Bitter-Tasting Children’s Oral Medicines. Chem. Pharm. Bull..

[53] Fritz F., Preissner R., Banerjee P. (2021). VirtualTaste: A Web Server for the Prediction of Organoleptic Properties of Chemical Compounds. Nucleic Acids Res..

